# Mapping QTLs Controlling Soybean Rust Disease Resistance in Chiang Mai 5, an Induced Mutant Cultivar

**DOI:** 10.3390/genes14010019

**Published:** 2022-12-21

**Authors:** Thongchai Chanchu, Tarika Yimram, Sompong Chankaew, Akito Kaga, Prakit Somta

**Affiliations:** 1 Department of Agronomy, Faculty of Agriculture at Kamphaeng Saen, Kamphaeng Saen Campus, Kasetsart University, Nakhon Pathom 73140, Thailand; 2 Department of Agronomy, Faculty of Agriculture, Khon Kaen University, Khon Kaen 40002, Thailand; 3 Institute of Crop Sciences, National Agriculture and Food Research Organization (NARO), 2-1-2 Kannondai, Tsukuba 305-8518, Ibaraki, Japan

**Keywords:** soybean, soybean rust, *Phakopsora pachyrhizi*, QTL

## Abstract

Soybean rust (SBR) caused by the fungus *Phakopsora pachyrhizi* is an important folia disease of soybean (*Glycine max*). In this study, we identified QTLs controlling SBR in Chiang Mai 5 (CM5), an SBR-resistant cultivar developed by induced mutation breeding. A recombinant inbred line (RIL) population of 108 lines developed from a cross between Sukhothai 2 (SKT2, a susceptible cultivar) and CM5 was evaluated for SBR resistance under field conditions in Thailand. QTL analysis for the resistance in the RIL population identified a single QTL, *qSBR18.1*, for resistance. *qSBR18.1* was mapped to a 212-kb region on chromosome 18 between simple sequence repeat markers Satt288 and sc21_3420 and accounted for 21.31–35.09% depending on the traits evaluated for resistance. The *qSBR18.1* interval overlapped with genomic regions containing *resistance to P. pachyrhizi 4* (*Rpp4*), a locus for SBR resistance. Three tightly linked genes, *Glyma.18G226250*, *Glyma.18G226300*, and *Glyma.18G226500*, each encoding leucine-rich repeat-containing protein, were identified as candidate genes for SBR resistance at the *qSRB18.1*. The *qSBR18.1* would be useful for breeding of SBR resistance.

## 1. Introduction

Soybean (*Glycine max* (L.) Merr.) is the most important legume crop in the world. Soybean seeds are a major source of protein and oil for human consumption and other uses. Soybean meal, a by-product of oil extraction, is used as the major protein source for animal feed production. In Thailand, soybean is the most important legume crop for the production of vegetable oil and animal feed. At present, Thailand uses about 4.0 million tons of soybean seeds annually, nearly all of which is imported [1]. During 1980–1990, the area under cultivation for soybean in the country was about 320,000 ha; however, at present, it is only 14,000 ha [1]. The shrinkage in soybean production in the country is due to several factors, including low yield. The average yield of soybean in Thailand during 2018–2020 was only 1670 kg/ha, about half that of leading soybean-producing countries such as Brazil and the United States [1]. The low yield is caused by several factors, including biotic and abiotic stresses. Among important biotic stresses of soybean in Thailand is soybean rust (SBR) disease caused by the fungus *Phakopsora pachyrhizi* Syd. The pathogen can infect all the aerial parts of the soybean plant but prefers the leaves. In general, the symptoms of SBR are tan to dark-brown or reddish-brown lesions with one or many erumpent and globose uredinia on leaves, especially the abaxial leaves [2]. In most cases, lesions are angular in shape and are restricted by leaf veins and associated with leaf chlorosis. In cases of heavy infection, the disease causes premature defoliation and early maturity, or failure to even reach maturity [3]. In Thailand, the disease occurs in the rainy season and causes yield reduction of up to 60% [4]. Therefore, enhancing resistance to SBR is a major objective of soybean breeding programs in Thailand. Several approaches have been used to develop soybean cultivars with high yield and resistance to SBR disease in the country. The soybean cultivar ‘Chiang Mai 5′ (CM5) has shown stable resistance to SBR disease under field conditions. CM5 is developed from Chiang Mai 60 (CM60), the most popular soybean cultivar in Thailand, by induced mutation using γ radiation [4]. CM5 expressed reddish-brown (RB) type 2 lesions against *P. pachyrhizi* infection under field and laboratory conditions and displayed RB type 3 lesions against eight isolates of *P. pachyrhizi* under laboratory conditions, while CM60 and Sukhothai 2 (SKT2) showed tan-type lesions against this pathogen, slow progress in disease development, and a low percentage of leaves affected by the disease [4]. 

So far, seven *resistance to P. pachyrhizi* (*Rpp*) loci conferring resistance to SBR disease have been reported and localized onto genetic linkage maps of soybean. *Rpp1*, *Rpp4*, and *Rpp6* were located on linkage group (LG) G (chromosome 18) [5,6,7,8,9,10,11,12,13,14,15,16], while *Rpp2*, *Rpp3*, *Rpp5*, and *Rpp7* were mapped onto LG J (chromosome 16), C2 (chromosome 6), N (chromosome 3), and L (chromosome 19), respectively [8,9,11,12,17,18,19,20,21]. Due to high variation in *P. pachyrhizi* races/isolates, a single *Rpp* locus/gene cannot provide durable and broad-spectrum resistance to SBR disease. For example, Akamatsu et al. [22] evaluated 59 *P. pachyrhizi* populations from various origins for their pathogenicity to 16 soybean differentials, including the ones containing *Rpp1*, *Rpp2*, *Rpp3*, *Rpp4*, and *Rpp5* genes, and found that *Rpp1* in PI 587880A and *Rpp5* were mostly effective against recent pathogen populations, because the differentials containing resistance genes *Rpp1*, *Rpp2*, *Rpp3*, and *Rpp4*, except for PI 587880A, which also contains *Rpp1*, expressed resistance to only 1.8–14%, 24–28%, 22%, and 36% of the *P. pachyrhizi* populations, respectively. Thus, identification of new loci and alleles conferring resistance to this disease is important. 

Although rust disease resistance in CM5 has been found to be associated with simple sequence repeat (SSR) markers Satt012, Satt288, and Satt472 on LG G [23], its position on the genetic linkage map is not yet known. Due to the fact that the resistance in CM5 is created by induced mutation, the gene/allele for the resistance in CM5 may be different from the *Rpp1*, *Rpp4*, and *Rpp6* that are found on LG G. Investigating the resistance gene(s) for the resistance in CM5 is interesting and will be useful for inducing soybean resistance to the rust disease. In this study, we identified a QTL controlling resistance to SBR disease in CM5 using recombinant inbred line (RIL) population. We showed that the QTL for the resistance in CM5 is localized on LG G, and its location appears to overlap with the *Rpp4*.

## 2. Materials and Methods

### 2.1. Plant Materials and DNA Extraction 

Recombinant inbred lines consisting of 108 lines were developed by a single seed descent method from a cross between Sukhothai 2 (male parent; hereafter called ‘SKT2’) and Chiang Mai 5 (female parent; hereafter called ‘CM5’). Both SKT2 and CM5 are commercial soybean cultivars from Thailand. SKT2 is susceptible to SBR disease whereas CM5 is resistant to the disease. The RILs have been used previously to investigate antagonistic pleiotropy effect of the *Ln* gene [24]. Details of DNA extraction and quantification have been described by Chanju et al. [24]. 

### 2.2. Evaluation of SBR Resistance

Evaluation of SBR resistance in the RILs was performed under natural field infection at Pang Da Royal Agricultural Station, Samerng, Chiang Mai, Thailand, in 2015. This station is located in a highland region and is a hotspot for SBR disease. It is always used for research and breeding for SBR disease. The RILs and their parents were sown in a randomized complete block design (RCBD) with two replicates during August–December (rainy season). In each replicate, each line was sown in a 5 m-long single row with 20 cm intra-row spacing and 50 cm inter-row spacing. CM5 was also sown around the experimental field as a guard row and source of natural inoculum. The resistance in CM5 is characterized by a low percentage of leaves affected by rust disease; therefore, 80 days after planting, the RILs and the parents were evaluated for resistance to the rust disease by visually scoring on individual plants using a scale of 1–5, where 1 = 0% leaves affected, 2 = 1–25% leaves affected, 3 = 26–50% leaves affected, 4 = 51–75% leaves affected, and 5 = 76–100% leaves affected. In each replicate, eight plants from each line were randomly selected and scored. The scoring was performed by a panel of three staff members. For the experiment in 2015, leaf chlorosis was also scored using a scale of 1–3, where 1 = no to slight leaf yellowness, 2 = moderate leaf yellowness, and 3 = extensive leaf yellowness. 

### 2.3. Analysis of Variance

The rust disease score and the rust leaf chlorosis of the RILs and the parents were subjected to analysis of variance (ANOVA) using software R-program 2.10.0.

### 2.4. DNA Marker Analysis

A total of 550 soybean SSR markers [11,25,26,27,28] were screened for polymorphism between SKT2 and CM5. For all the primers, except those reported by Watanabe et al. [28], a polymerase chain reaction (PCR) mixture in a total volume of 10 µL, containing 2.0 µL of 2.0 ng/µL of template DNA, 1.0 µL of 10 × Taq buffer, 0.8 µL of 25 mM MgCl_2_, 2.0 µL of 1.0 mM dNTPs, 0.2 µL of 5 unit of *Taq* DNA polymerase (Fermentas, Lithuania), 1.0 µL of 0.5 pmol of forward primer, 1.0 µL of 0.5 pmol of reverse primer, and 2.0 µL of deionized water was prepared. Amplification was performed in a GeneAmp PCR 9700 System thermocycler (Applied Biosystems, Foster City, CA, USA) programmed as follow: 94 °C for 2 min followed by 35 cycles of 94 °C for 30 s, 47 °C for 30 s, 72 °C for 1 min, and 72 °C for 10 min. The PCR products were separated on 5% denaturing polyacrylamide gels and visualized by silver staining. After marker screening, markers showing polymorphism between the parents were used to analyze and genotype the DNA of the RILs. For the primers reported by Watanabe et al. [28], the method of genotyping using a universal fluorescently labelled (UFL) primer described by these authors was applied. Three fluorescent dyes, 6-FAM, HEX, and NED (Applied Biosystems, Foster City, CA, USA), were used.

### 2.5. Linkage Map and QTL Analyses

A linkage map was constructed for the RIL population using the software QTL IciMapping 4.1 [29]. The markers were grouped with a minimum logarithm of the odds (LOD) score of 3.0 and subsequently ordered using the REcombination Counting and ORDering (RECORD) algorithm [30]. The map distance was calculated using Kosambi’s mapping function [31]. 

The DNA marker(s) associated with rust disease and leaf chlorosis were identified by single marker analysis using the likelihood ratio test method by the software QTL IciMapping 4.1. 

QTLs conditioning the percentage of leaves affected by rust disease and leaf chlorosis caused by the disease were located onto the linkage map by inclusive composite interval mapping [32] using the software QTL IciMapping 4.1. Significant LOD score threshold for the QTL was determined by running 1000-time permutation tests at the probability level of 95%. 

### 2.6. Identification of Candidate Gene(s) for Resistance 

Based on results from QTL analysis, sequences of markers flanking major QTL for rust resistance were subjected to BLASTN against the whole genome sequence of soybean cultivar ‘William 82′ [33]; https://phytozome-next.jgi.doe.gov (accessed on 25 September 2022). Genes with structure or function related to disease resistance residing within the genome region covering the flanking markers were selected as candidate genes for rust resistance.

## 3. Results

### 3.1. Rust Disease Variation in the RIL Population and Parents

**Figure 1:**
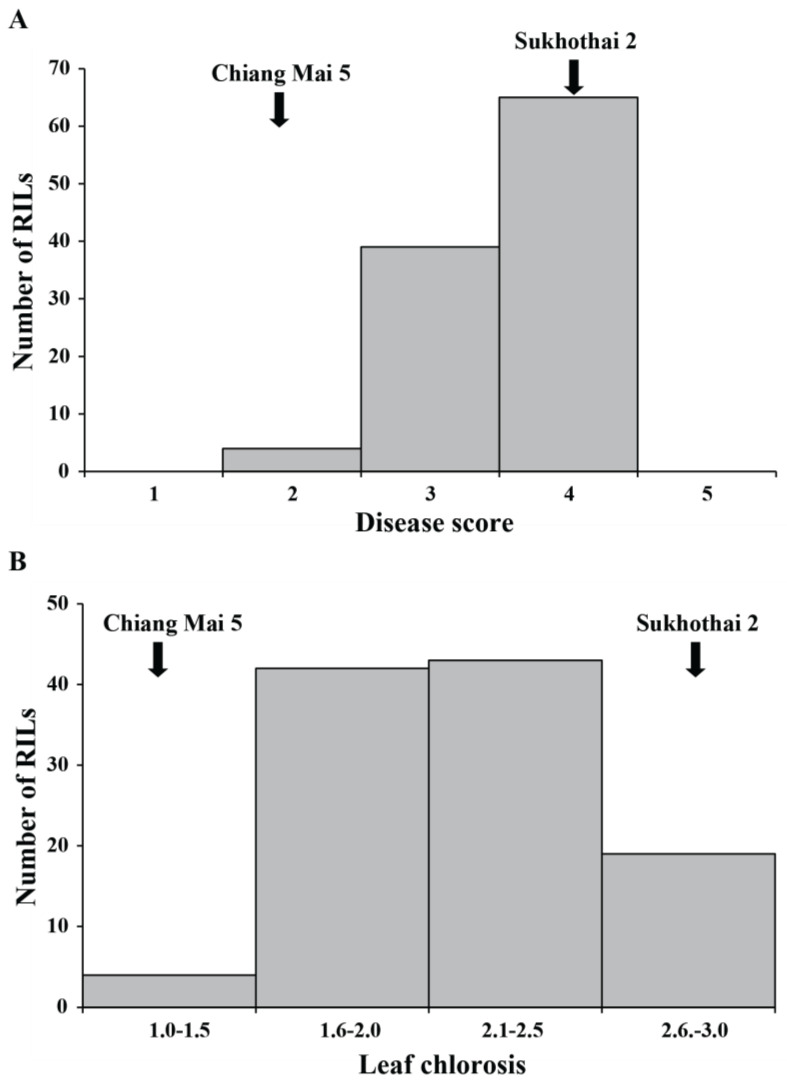
Frequency distribution of leaf rust disease score (**A**) and leaf chlorosis (**B**) caused by *Phakopsora pachyrhizi* in 108 RIL lines derived from a cross between Sukhothai 2 and Chiang Mai 5.

The RIL population of the cross SKT2 × CM5 was evaluated for SBR resistance in the two years of study. In 2015, the disease severity scores in the RILs ranged from 2.47 to 4.50 with a mean of 3.60, whereas the scores in SKT2 and CM5 were 4.61 and 2.49, respectively. The leaf chlorosis scores in the RILs ranged from 1.67 to 2.83 with a mean of 2.16, whereas the scores in SKT2 and CM5 were 1.66 and 2.75, respectively. Frequency distribution of the disease severity and leaf chlorosis scores of the RIL population are shown Figure 1. Most of the RILs were moderately resistant (disease severity score of 3 and leaf chlorosis score of 1.6–2.5). ANOVA revealed significant difference in the leaf disease and leaf chlorosis scores among the RILs (Tables S1 and S2).

### 3.2. Linkage Map and QTL for Rust Disease Resistance 

Of the 550 SSR markers screened for polymorphism between SKT2 and CM5, 130 revealed clear polymorphisms under our PCR conditions. These SSRs together with the *Ln* locus controlling leaf shape were used to construct a genetic linkage map for the RIL population. The map comprised 28 LGs (Figure S1) with six markers—Sat_160, Satt362, Satt192, Satt481, Satt537, and Satt546—remaining unlinked. The number of markers per LG ranged from 2 to 47. Several LGs contained 2 or 3 markers. As expected, LG G contained the highest number of markers, and the average distance between markers was 5.89 cM. 

Single marker analysis using the likelihood ratio test method revealed that 28 and 23 markers were significantly associated with rust disease and leaf chlorosis, respectively (Tables S3 and S4). All the markers were on the LG G and accounted for less than 10% of the total variation of the trait.

**Figure 2:**
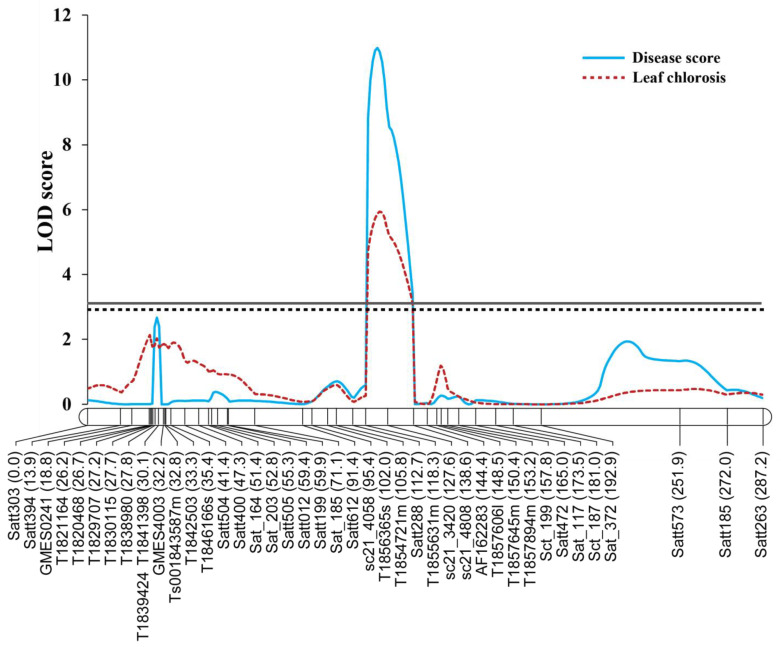
A logarithm of odds (LOD) score plot of QTLs controlling leaf rust disease score and leaf chlorosis caused by *Phakopsora pachyrhizi* in 108 RILs derived from a cross between Sukhothai 2 and Chiang Mai 5. Complete and dotted lines horizontal to the *x*-axis are the LOD threshold for the leaf rust disease score and leaf chlorosis, respectively. The number in parenthesis after a marker name indicates the marker location (in centimorgan).

QTL for rust resistance was located onto the linkage map using the ICIM method. The results of the QTL analysis are summarized in Table 1. For the disease severity score, a single QTL was identified for the trait (Figure 2). The *qSBR18.1* was located at 123 cM between markers T001855631m and sc21_3420 on LG G (chromosome 18). It accounted for 35.09% of the total disease score variation. At this QTL, allele(s) from CM5 reduced the disease severity. In the of case of the leaf chlorosis score, a single QTL was identified at nearly the same position as the disease severity score, with it being at 124 cM and in the same interval with the QTL identified for the disease severity score (Figure 2). It explained 21.31% of the total leaf chlorosis variation. Allele(s) from CM5 decreased leaf chlorosis. Since the QTLs for the disease score variation and leaf chlorosis were localized at nearly the same position, we considered them as the same locus. 

### 3.3. Candidate Genes for the qSBR18.1 

**Figure 3:**
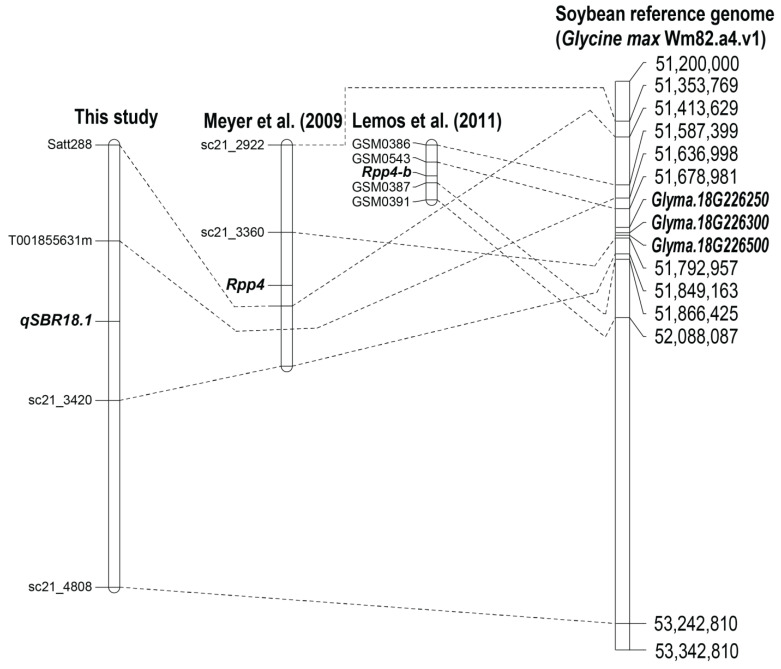
A comparative map showing the relationship between the QTL *qSBR18.1* controlling resistance to soybean rust disease caused by *Phakopsora pachyrhizi* detected in this study, and the *Rpp4* locus conferring resistance to soybean rust disease detected previously. Physical location of markers and candidate genes on the soybean reference genome are also included on the right of the map [12,14].

The QTL *qSBR18.1* appeared to be the major locus conferring SBR resistance in CM5, and we identified candidate genes for this QTL. A BLASTN search against the soybean reference genome sequence (*G. max* Wm82.a4.v1) revealed that the markers T001855631m and sc21_3420 were about 212.17 kb apart on chromosome 18 at the positions 51,636,998 bp and 51,849,163 bp, respectively (Figure 3). There were 13 predicted genes in this 212.17 kb region (Table S5). Among these genes, three tightly linked genes, including *Glyma.18G226250* (Gm18: 51,753,139 bp..51,765,121 bp), *Glyma.18G226300* (Gm18: 51,765,121 bp..51,782,390 bp), and *Glyma.18G226500* (Gm18: 51,789,555 bp..51,832,726 bp), each encoding leucine-rich repeat (LRR)-containing protein, were selected as candidate genes for the SBR resistance at *qSRB18.1*. These three genes were clustered in a small region of only 79.5 kb (51,753,139–51,832,726).

## 4. Discussion

At present, seven *Rpp* genes, *Rpp1*–*Rpp7*, have been identified for rust disease resistance in soybean. In this study, we molecularly mapped a major QTL, *qSBR18.1*, conferring rust resistance in CM5, an induced mutant soybean cultivar. *qSBR18.1* was located on chromosome 18 with the genes *Rpp1*, *Rpp4*, and *Rpp6* and appeared to be the same locus with *Rpp4*. A fine mapping study using soybean accession PI 459025B as the source of SBR resistance showed that the *Rpp4* was located between SSR markers sc21_3360 and Satt288 [14]. Later, an *Rpp* gene controlling SBR resistance in accession PI 423972 was mapped onto chromosome 18 between single nucleotide polymorphism (SNP) markers GSM0543 and GSM0387 [12]. This gene appeared to be allelic to *Rpp4* and was designated ‘*Rpp4-b*’ [12]. In our study, the *qSBR18.1* was mapped between the SSR markers T001855631m and sc21_3420. Comparative genome analysis demonstrated that the *qSBR18.1* interval overlapped with the *Rpp4* and *Rpp4-b* regions (Figure 3). All seven *Rpp* genes were identified in soybean cultivars/accessions with natural variations (see [3] for review). In contrast to those genes, *qSBR18.1* was identified in CM5, which gained the resistance from induced mutation. Thus, it is very likely that *qSBR18.1* is different from *Rpp4* and *Rpp4-b*, or they are at least the same locus but different alleles. 

Among the seven *Rpp* genes, candidate genes have been identified for the *Rpp1*, *Rpp2*, and *Rpp4* genes in which genes encoding proteins with LRR domain(s) appeared to be involved in the resistance. The *Rpp1* contained three homologs of the nucleotide binding site–LRR (NBS–LRR) gene, each encoding N-terminal ubiquitin-like protease 1 that confers resistance [34]. *Rpp2* harbored ten toll/interleukin-1 receptor (TIR)- NBS–LRR genes [19]. *Rpp4* harbored coiled-coil (CC)–NBS–LRR genes controlling resistance [14]. In the plant immune system, specificity determinants of effector-triggered immunity are controlled by resistance (*R*) genes. Most of the *R* genes encode proteins that contain an NBS–LRR domain. The NBS–LRR proteins are involved in the recognition of pathogen effectors (avirulence (Avr) proteins) that are believed to confer virulence function in the absence of the cognate *R* gene [35]. In our study, based on the most recent annotation of the reference soybean genome sequence (*G. max* Wm82.a4.v1), *Glyma.18G226250*, *Glyma.18G226300*, and *Glyma.18G226500*, each producing proteins possessing an LRR domain, were identified as the candidate genes at *qSBR18.1*. *Glyma.18G226300* and *Glyma.18G226500* were previously identified for the *Rpp4-b* using a previous soybean reference genome annotation [12]. A BLASTP search of the proteins encoded by these soybean genes using the *Arabidopsis* Information Resource (TAIR) database revealed that *Glyma.18G226250*, *Glyma.18G226300*, and *Glyma.18G226500* showed the best hit with *AT4G27190* (E-value = 1 × 10^−56^ and identities = 27%), *AT4G26090* (E-value = 7 × 10^−5^ and identities = 26%), and *AT4G27220* (E-value = 1 × 10^−55^ and identities = 26%), respectively, with *AT4G27190* and *AT4G27220* each encoding NB-ARC domain-containing protein. The NB-ARC domain is a functional ATPase domain, and its nucleotide-binding state is proposed to regulate the activity of the disease resistance protein [36]. *AT4G26090* (*resistant to Pseudomonas syringae 2* (*RPS2*)) produces a cell membrane protein with LRR, leucine zipper, and P loop domains that provides resistance to *P. syringae* [37]. Additional studies are necessary to determine which one of these genes or which combination of genes control SBR resistance in CM5. Nonetheless, since CM5 is derived from an induced mutation, the MutMap technique [38] can be used to effectively identify the causative gene controlling rust resistance in CM5.

## Figures and Tables

**Table 1:** QTLs detected for soybean rust disease caused by *Phakopsora pachyrhizi* in 108 RILs derived from a cross between Sukhothai 2 and Chiang Mai 5. The QTLs were detected by the inclusive composite interval mapping method.

Trait	QTL Name	Linkage Group	Position (cM)	LOD Score	Flanking Markers	Percentage of Variance Explained	Additive Effect
Rust disease	*qSBR* *18.1*	G	123	10.90	T001855631m—sc21_3420	37.55	0.36
Rust leaf chlorosis	*qSBR* *18.1*	G	124	5.94	T001855631m—sc21_3420	21.74	1.86

## Data Availability

Not applicable.

## Supplementary Materials

The following supporting information can be downloaded at: https://www.mdpi.com/article/10.3390/genes14010019/s1, Figure S1: SSR-based linkage map constructed from the soybean RIL population of 108 lines of the cross between Sukhothai 2 and Chiang Mai 5.; Table S1: Results of analysis of variance of rust disease score of the 110 genotypes (108 recombinant inbred lines and their parents (Sukhothai 2 and Chiang Mai 5)) that were evaluated for resistance to *Phakopsora pachyrhizi*.; Table S2: Results of analysis of variance of rust leaf chlorosis of the 110 genotypes (108 recombinant inbred lines and their parents (Sukhothai 2 and Chiang Mai 5)) that were evaluated for resistance to *Phakopsora pachyrhizi*.; Table S3: DNA markers showing association with rust disease score caused by *Phakopsora pachyrhizi* in 108 recombinant inbred lines derived from a cross between Sukhothai 2 and Chiang Mai 5. The markers were identified by single marker analysis using the likelihood ratio test.; Table S4: DNA markers showing the association with rust leaf chlorosis score caused by *Phakopsora pachyrhizi* in 108 recombinant inbred lines derived from a cross between Sukhothai 2 and Chiang Mai 5. The markers were identified by single marker analysis using the likelihood ratio test.; Table S5: Details of 13 annotated genes located between markers T001855631m and sc21_3420 flanking the QTL *qSBR18.1* on the soybean chromosome 18 controlling resistance to soybean rust disease caused by *Phakopsora pachyrhizi*, detected in 108 RILs derived from a cross between Sukhothai 2 and Chiang Mai 5.
