# Supplementary material for: Mapping QTLs Controlling Soybean Rust Disease Resistance in Chiang Mai 5, an Induced Mutant Cultivar
Source: Genes (Basel). 2022 Dec 21;14(1):19. doi: 10.3390/genes14010019 (PMC9858275; doi:10.3390/genes14010019)

Figure S1. SSR-based linkage map constructed from the soybean RIL population of 108 lines of the cross between Sukhothai 2 and Chiang Mai 5.

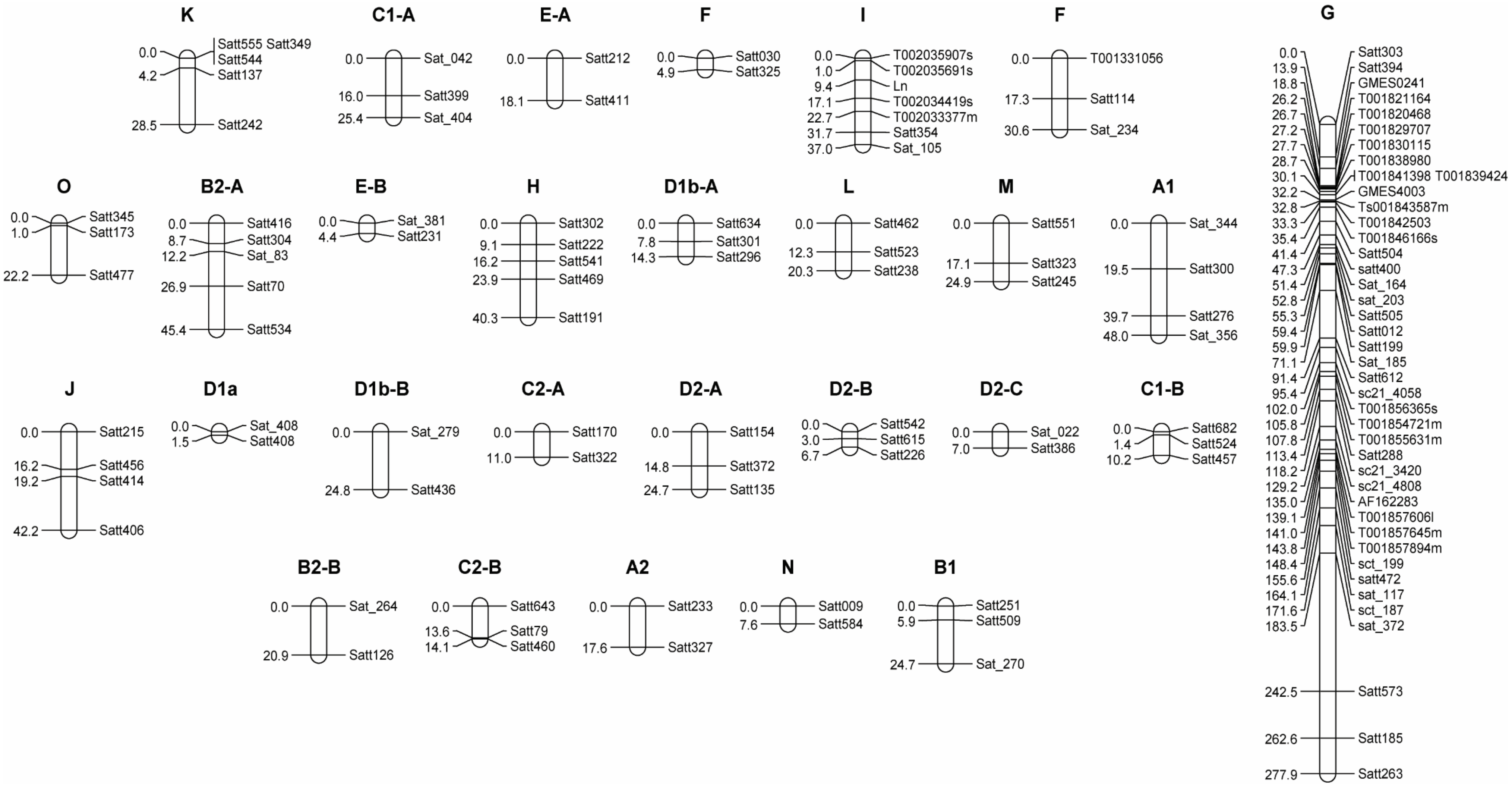

Supplement: Supplementary file 1 [file genes-14-00019-s001.zip › Figure S1.pdf]
