# Supplementary material for: Mapping QTLs Controlling Soybean Rust Disease Resistance in Chiang Mai 5, an Induced Mutant Cultivar
Source: Genes (Basel). 2022 Dec 21;14(1):19. doi: 10.3390/genes14010019 (PMC9858275; doi:10.3390/genes14010019)
Supplement: Supplementary file 1 [file genes-14-00019-s001.zip › Table S1.pdf]

Table S1. Details of 13 annotated genes locating between markers T001855631m and sc21_3420 flanking the QTL *qSBR18.1* on the soybean chromosome 18 controlling resistance to soybean rust disease caused by *Phakopsora pachyrhizi* detected in 108 RILs derived from a cross between Sukhothai 2 and Chiang Mai 5.

Gene	Position on chromosome 18	Protein
Glyma.18G225400	51639851..51640752	BON1-ASSOCIATED PROTEIN 1-RELATED
Glyma.18G225500	51640929..51645812	RING FINGER DOMAIN-CONTAINING protein
Glyma.18G225600	51649274..51650627	PENTATRICOPEPTIDE (PPR) REPEAT-CONTAINING PROTEIN
Glyma.18G225700	51651369..51656265	GLYCOGEN SYNTHASE KINASE-3 ALPHA // SHAGGY-RELATED PROTEIN KINASE BETA-RELATED
Glyma.18G225800	51673815..51680882	homeobox-leucine zipper protein (HD-ZIP)
Glyma.18G225850	51703421..51708090	RNA-binding protein
Glyma.18G225900	51705916..51712814	METHYLTRANSFERASE // METHYLTRANSFERASE PMT13-RELATED
Glyma.18G226200	51733771..51741540	METHYLTRANSFERASE
Glyma.18G226250	51753139..51765121	LEUCINE-RICH REPEAT-CONTAINING PROTEIN
Glyma.18G226300	51765121..51782390	LEUCINE-RICH REPEAT-CONTAINING PROTEIN
Glyma.18G226400	51783146..51786175	Protein of unknown function
Glyma.18G226500	51789555..51832726	LEUCINE-RICH REPEAT-CONTAINING PROTEIN
Glyma.18G226551	51843008..51843128	***No homologs were found for this gene***
