# Supplementary material for: Mapping QTLs Controlling Soybean Rust Disease Resistance in Chiang Mai 5, an Induced Mutant Cultivar
Source: Genes (Basel). 2022 Dec 21;14(1):19. doi: 10.3390/genes14010019 (PMC9858275; doi:10.3390/genes14010019)
Supplement: Supplementary file 1 [file genes-14-00019-s001.zip › Supplementary data/Table S1.pdf]

Table S1. Results of analysis of variance of rust disease score of the 110 genotypes (108 RILs and their parents (Sukhothai 2 and Chiang Mai 5)) that were evaluated for resistance to *Phakopsora pachyrhizi*.

Source of variance	Degree of freedom	Sum of square	Mean square	F-test	Probability value
Replication	1	0.021	0.021	0.125	0.7244
Line	109	75.037	0.688	4.043	1.202e-12
Error	109	18.562	0.170		
Total	219	93.620			
