# Supplementary material for: Mapping QTLs Controlling Soybean Rust Disease Resistance in Chiang Mai 5, an Induced Mutant Cultivar
Source: Genes (Basel). 2022 Dec 21;14(1):19. doi: 10.3390/genes14010019 (PMC9858275; doi:10.3390/genes14010019)
Supplement: Supplementary file 1 [file genes-14-00019-s001.zip › Supplementary data/Table S2.pdf]

Table S2. Results of analysis of variance of rust leaf chlorosis of the 110 genotypes (108 RILs and their parents (Sukhothai 2 and Chiang Mai 5)) that were evaluated for resistance to *Phakopsora pachyrhizi*.

Source of variance	Degrees of freedom	Sum of square	Mean square	F-test	Probability value
Replication	1	0.011	0.011	0.089	0.7667
Line	109	29.524	0.271	2.271	1.254e-05
Error	109	13.001	0.119		
Total	219	42.536			
