# Supplementary material for: Mapping QTLs Controlling Soybean Rust Disease Resistance in Chiang Mai 5, an Induced Mutant Cultivar
Source: Genes (Basel). 2022 Dec 21;14(1):19. doi: 10.3390/genes14010019 (PMC9858275; doi:10.3390/genes14010019)
Supplement: Supplementary file 1 [file genes-14-00019-s001.zip › Supplementary data/Table S3.pdf]

Table S3. DNA markers showing association with rust disease score caused by *Phakopsora pachyrhizi* in 108 recombinant inbred lines derived from a cross between Sukhothai 2 and Chiang Mai 5. The markers were identified by single marker analysis using likelihood ratio test.

Marker name	Linkage group	Position on linkage group (centimorgan)	LOD value	Percentage of variance explained (%)	Additive effect
T001821164	G	26.19	3.1922	1.0159	0.2052
T001830115	G	27.68	3.2524	1.0337	0.2066
T001838980	G	28.66	3.6009	1.1361	0.216
T001841398	G	30.14	3.8012	1.1943	0.2213
T001839424	G	30.14	3.8012	1.1943	0.2213
GMES4003	G	32.19	4.2525	1.3235	0.2329
Ts001843587m	G	32.75	4.2822	1.3320	0.2339
T001842503	G	33.34	3.9506	1.2373	0.2254
T001846166s	G	35.38	5.4398	1.6519	0.2608
Satt504	G	41.39	5.1045	1.5608	0.2564
satt400	G	47.27	4.7148	1.4534	0.246
Sat_164	G	51.40	4.5281	1.4012	0.2446
sat_203	G	52.83	6.1943	1.8521	0.2802
Satt505	G	55.26	5.7467	1.7342	0.2687
Satt012	G	59.39	6.0713	1.8199	0.2747
Satt199	G	59.88	5.4793	1.6625	0.2631
Satt612	G	91.38	4.7823	1.4721	0.247
sc21_4058	G	95.39	6.6514	1.9703	0.2848
T001856365s	G	102.02	8.7699	2.4890	0.3228
T001854721m	G	105.80	8.5885	2.4464	0.3261
T001855631m	G	107.84	9.1640	2.5804	0.332
Satt288	G	113.42	9.1130	2.5687	0.3312
sc21_3420	G	118.22	9.2119	2.5915	0.3259
sc21_4808	G	129.19	3.5536	1.1223	0.2147
T001857606l	G	139.13	4.3437	1.3493	0.2357
T001857645m	G	141.03	4.2283	1.3166	0.2322
T001857894m	G	143.81	3.3881	1.0737	0.2098
sct_199	G	148.41	3.2131	1.0221	0.2054
