# Supplementary material for: Mapping QTLs Controlling Soybean Rust Disease Resistance in Chiang Mai 5, an Induced Mutant Cultivar
Source: Genes (Basel). 2022 Dec 21;14(1):19. doi: 10.3390/genes14010019 (PMC9858275; doi:10.3390/genes14010019)
Supplement: Supplementary file 1 [file genes-14-00019-s001.zip › Supplementary data/Table S4.pdf]

Table S4. DNA markers showing association with rust leaf chlorosis score caused by *Phakopsora pachyrhizi* in 108 recombinant inbred lines derived from a cross between Sukhothai 2 and Chiang Mai 5. The markers were identified by single marker analysis using likelihood ratio test.

Marker name	Linkage group	Position on linkage group (centimorgan)	LOD value	Percentage of variance explained (%)	Additive effect
T001838980	G	28.66	3.1052	1.0296	0.1273
T001841398	G	30.14	3.0935	1.0259	0.1269
T001839424	G	30.14	3.0935	1.0259	0.1269
GMES4003	G	32.19	3.9645	1.2910	0.1424
Ts001843587m	G	32.75	3.7934	1.2396	0.1397
T001842503	G	33.34	3.6134	1.1853	0.1366
T001846166s	G	35.38	4.3858	1.4156	0.1494
Satt504	G	41.39	3.9405	1.2837	0.1439
satt400	G	47.27	3.5166	1.1559	0.1358
Sat_164	G	51.4	3.4628	1.1394	0.1366
sat_203	G	52.83	3.7883	1.2381	0.1418
Satt505	G	55.26	3.3438	1.1031	0.1327
Satt012	G	59.39	3.8528	1.2575	0.1413
Satt199	G	59.88	3.6950	1.2100	0.1389
Satt612	G	91.38	3.3211	1.0962	0.1319
sc21_4058	G	95.39	3.8102	1.2448	0.1401
T001856365s	G	102.02	4.7060	1.5089	0.1556
T001854721m	G	105.8	4.9474	1.5784	0.1621
T001855631m	G	107.84	4.2122	1.3645	0.1494
Satt288	G	113.42	5.0577	1.6099	0.1623
sc21_3420	G	118.22	5.1954	1.6491	0.1609
T001857606l	G	139.13	3.9965	1.3005	0.1432
T001857645m	G	141.03	4.3388	1.4018	0.1483
